# Endoscopic thyroidectomy via the submental approach: a balanced approach to aesthetics, safety, and recovery- a case series study

**DOI:** 10.3389/fendo.2025.1715390

**Published:** 2026-01-12

**Authors:** Wu Li, Wenkai Li, Peng Wu, Hui Li, Yulong Tang, Xiaohua Song, Xiaowei Peng, Shiwei Zhou

**Affiliations:** Department of Thyroid Surgery, The Affiliated Cancer Hospital of Xiangya School of Medicine, Central South University/Hunan Cancer Hospital, Changsha, Hunan, China

**Keywords:** endoscopic thyroidectomy via the submental approach, papillary thyroid carcinoma, surgical outcomes, cosmetic satisfaction, quality of life, surgical feasibility

## Abstract

**Objective:**

To overcome certain anatomical limitations of transoral vestibular thyroidectomy, we introduced endoscopic thyroidectomy via the submental approach. During subsequent clinical practice, additional advantages of the submental approach were identified, further enhancing its feasibility and clinical applicability. This study aimed to evaluate the safety, efficacy, and postoperative outcomes of this technique in thyroid surgery.

**Methods:**

We retrospectively analyzed patients who underwent endoscopic thyroidectomy via the submental approach at Hunan Cancer Hospital between March and September 2023. Perioperative outcomes, complications, and postoperative quality of life were assessed using validated clinical indicators and the Thyroid Cancer-Specific Quality of Life questionnaire.

**Results:**

A total of 50 patients (27 males, 23 females; mean age 41.66 ± 9.70 years) were included. The mean operative time was 93.61 ± 21.78 minutes for unilateral thyroidectomy and 137.39 ± 27.76 minutes for total thyroidectomy. The mean pain score at 48 hours postoperatively was 1.56 ± 0.61, and the mean postoperative hospital stay was 2.84 ± 0.81 days. The mean aesthetic satisfaction score was 4.28 ± 0.67 (1–5, with higher scores indicating better satisfaction). The mean quality-of-life score was 10.23 ± 5.33 (0–130, with lower scores indicating better quality of life). No surgical site infections occurred. Transient hypoparathyroidism occurred in 14.8% of patients, and transient recurrent laryngeal nerve palsy occurred in 4.00%. The mean follow-up duration was 22 months, and no cases of recurrence or distant metastasis were observed during the follow-up period.

**Conclusions:**

Endoscopic thyroidectomy via the submental approach effectively balances cosmetic outcomes, surgical trauma, oncological integrity, and surgical feasibility, providing a promising alternative for endoscopic thyroidectomy. Further prospective, multicenter studies are needed to validate its long-term safety and broader clinical applicability.

**Clinical trial registration:**

https://www.chictr.org.cn, identifier ChiCTR2400089684.

## Introduction

With the growing demand for scarless surgery and continuous advancements in minimally invasive techniques, endoscopic thyroid surgery (ETS) has evolved as an important alternative to conventional open thyroidectomy (1, 2) (3). Since the first report of endoscopic right thyroid lobectomy by Hüscher et al. in 1997 (4), various remote-access approaches—such as the transaxillary, breast, chest, and oral vestibular routes—have been developed to improve cosmetic outcomes while ensuring oncological safety (5–7).

A significant milestone was reached in 2009, when Benhidjeb et al. introduced the concept of totally transoral video-assisted thyroidectomy, pioneering the application of natural orifice transluminal endoscopic surgery (NOTES) to thyroid surgery (8). Building upon this concept, the transoral endoscopic thyroidectomy vestibular approach (TOETVA) has gained widespread clinical adoption. TOETVA fully conforms to the NOTES principle, leaving no visible scar, minimizing flap dissection, and offering direct midline access for central neck dissection (1, 9, 10).

While TOETVA has gained popularity as a scar-free approach, its applicability is limited in certain patient populations. Specifically, patients with a high thyroid cartilage notch (11), mandibular prominence, oral mucosal fibrosis, small oral cavity, or prior prosthetic implants may face significant technical challenges when undergoing TOETVA. The anatomical constraints in these individuals make trocar placement and instrument maneuverability difficult, increasing the risk of complications such as mental nerve injury or excessive soft tissue traction (12, 13).

To address these anatomical limitations, we propose a novel approach: the endoscopic thyroidectomy via trans-submental approach (ETSA). This technique places both the observation and operative ports in the submental region, specifically in the natural skin fold approximately 1 cm below the chin, which helps avoid the anatomical restrictions encountered with the TOETVA.

In clinical practice, we have observed that the submental approach not only overcomes the anatomical limitations of the transoral route but also offers advantages such as a hidden incision, minimal trauma, thorough central lymph node dissection and simplified operation. As the application of this technique has expanded, it has demonstrated excellent results not only in patients with special anatomical considerations but also in routine patients, showing good surgical outcomes and cosmetic results. Therefore, this case series aims to assess the efficacy of the ETSA and further explore its potential to improve postoperative quality of life, enhance cosmetic satisfaction, and ensure oncological safety.

## Methods

We retrospectively analyzed the data of patients who underwent ETSA at the Department of Thyroid Surgery, Hunan Cancer Hospital, from March to September 2023. All surgeries were performed by the same surgical team. The surgical extent for each patient was determined based on the results of a preoperative multidisciplinary team (MDT) discussion.

### Inclusion criteria

1. Patients aged 18 years or older;
2. Preoperative needle biopsy or intraoperative pathology confirmed the diagnosis of papillary thyroid carcinoma requiring central compartment lymph node dissection;
3. Tumor maximum diameter ≤ 4.0 cm;
4. No abnormalities detected on preoperative laryngoscopy.
5. No parathyroid diseases.

### Exclusion criteria

1. Conversion from endoscopic to conventional open thyroidectomy (COT) surgery;
2. History of neck surgery or radiotherapy;
3. Patients who were unfit for surgery;
4. Hyperthyroidism or hypothyroidism;
5. Tumor invasion of adjacent structures, such as the trachea, esophagus and recurrent laryngeal nerve (RLN);
6. Lateral neck or distant metastasis;
7. Patients who were lost to follow-up or declined to participate in the study.

The study was approved by the Medical Ethics Committee of the Hunan Cancer Hospital. In addition, this study was registered at Chinese Clinical Trial Registry (UIN: ChiCTR2400089684, https://www.chictr.org.cn) in accordance with the World Medical Association’s Declaration of Helsinki, 2013. All patients agreed that their personal statistics for clinical research and signed informed consent. This study has been reported in line with the STROCSS criteria (14).

### Surgical procedure

The patient was placed in the supine position with the head slightly extended and fixed, and a pillow was placed under the shoulders. General anesthesia was induced, and routine disinfection and draping were performed.

**Figure 1:**
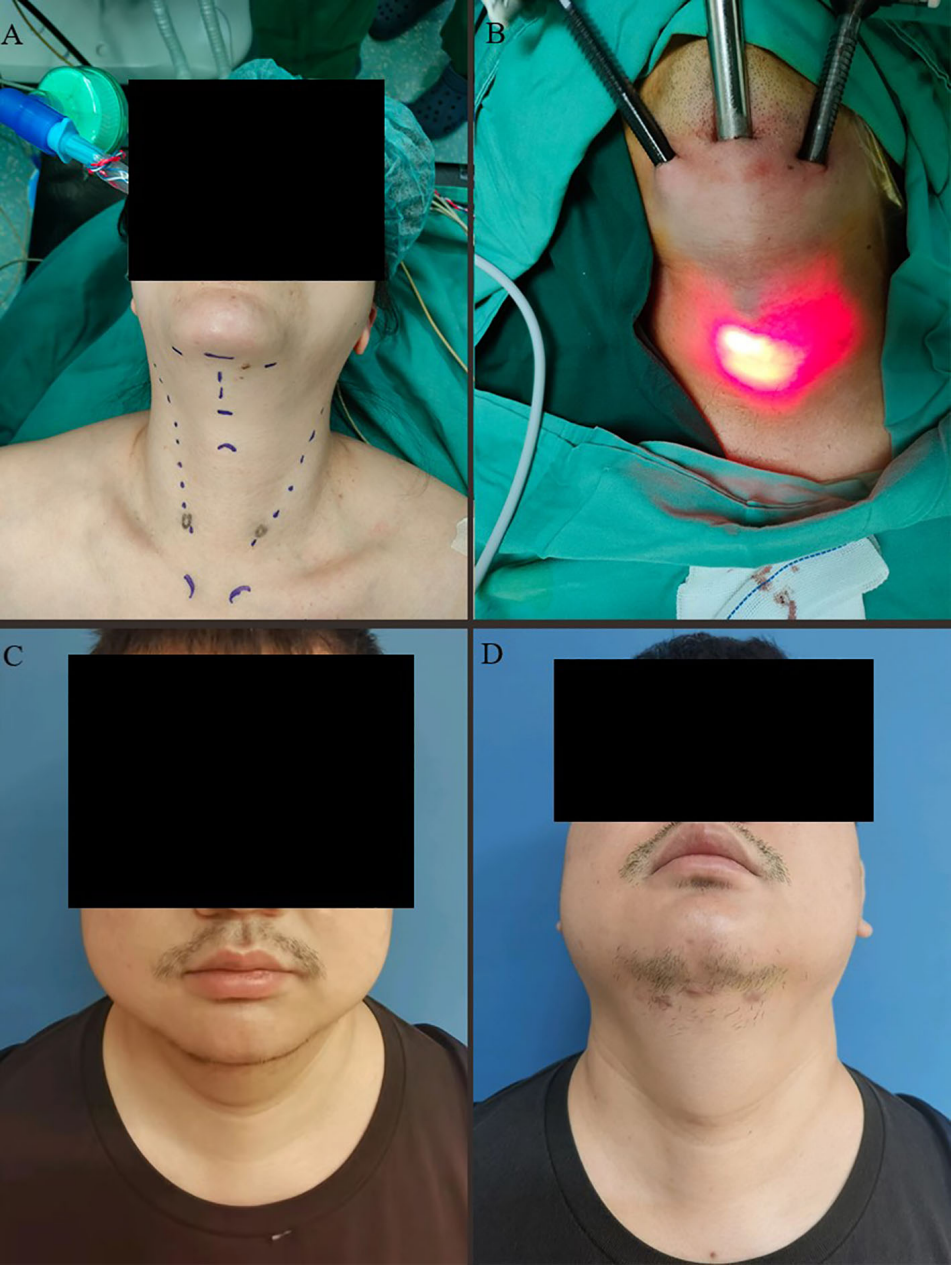
**(A)** A woman chose ETHSI surgery due to concerns about potential displacement or fracture of prosthesis implants. The blue marking line illustrates the incision and anatomical markers before surgery. **(B)** Trocar placement positions for observation and manipulation ports in a male patient. **(C, D)** Images depicting the frontal and head-up positions of a male patient one month after surgery.

5 mL of expansion solution (500 mL saline with 0.05 mg adrenaline) was injected to create the operative space. A transverse incision of approximately 0.8 cm in length was made at the skin fold below the chin. The incision was made through the skin and subcutaneous tissue to the strap muscles of the neck. Bilateral incisions were made 4 cm lateral to the midline incision, approximately 5 mm in length. A 10-mm trocar was inserted through the midline incision and 5-mm trocars were inserted bilaterally (**Video 1**). Carbon dioxide was insufflated to maintain a pressure of 6–8 mmHg. The operative space was created using an electrocautery hook along the deep surface of the platysma muscle and the superficial layer of the deep cervical fascia (**Figures 1A, B**).

An electrocautery hook or harmonic scalpel was used to create the working space. The strap muscles were separated along the midline using the same instrument, extending superiorly to the thyroid cartilage notch and inferiorly to the sternal notch. The thyroid gland was exposed, and the strap muscles were suspended with a 3–0 silk suture. A lymphatic tracer (mitoxantrone hydrochloride injection) was administered at a dose of 0.1 mL per thyroid lobe. The middle thyroid vein was severed with ultrasonic scalpel. The upper portion of the sternothyroid muscle was severed to expose the cricothyroid space and to fully “bare” the upper pole of the thyroid gland. The upper pole vessels were coagulated using an ultrasonic scalpel, and the upper parathyroid glands was carefully preserved *in situ*. The RLN was identified and preserved throughout the procedure. The inferior parathyroid gland was carefully identified and either preserved *in situ* or autotransplanted as appropriate. The thyroid gland was excised, and central neck lymph node dissection was performed concurrently. Intraoperative monitoring of the vagus and RLN signals was done to ensure nerve integrity and continuity.

The wound was irrigated with sterile distilled water, and hemostasis was confirmed. A small drainage tube was placed at the surgical site. Finally, the three small incisions were closed with layers of fine cosmetic sutures.

### Postoperative management

Ice application for the first 24 hours, followed by the use of a neck and submental compression band. The patient was allowed to consume liquid diet 6 hours postoperatively. The drainage tube was removed when the daily drainage volume was <15 mL. All patients received thyroid-stimulating hormone (TSH) suppression therapy, and regular outpatient follow-up was arranged to monitor recovery and detect any signs of recurrence.

Postoperative laryngoscopy was routinely performed for all patients before discharge. This standardized protocol allowed for objective assessment of RLN function and detection of both symptomatic and asymptomatic vocal cord palsies.

Postoperative quality of life (QoL) was assessed using the validated Chinese version of the Thyroid Cancer-specific Quality of Life (THYCA-QoL) questionnaire, following prior consent and authorization for its use. The THYCA-QoL instrument has a mean scoring range from 0 to 130, with lower scores indicating better postoperative QoL.

Cosmetic satisfaction was evaluated using a five-point Likert scale: 1 (very dissatisfied), 2 (dissatisfied), 3 (neutral), 4 (satisfied), and 5 (very satisfied).

Postoperative pain was assessed using a Visual Analog Scale (VAS) at 48 hours after surgery, with scores ranging from 0 (no pain) to 10 (worst imaginable pain).

Postoperative complications, such as RLN palsy, hypoparathyroidism, hematoma, chyle leakage, bleeding and infection, were recorded and evaluated according to predefined criteria. A complete definition of each outcome, along with its measurement method and evaluation timeframe, was provided in Supplementary **Table 1**.

**Table 1:** Demographic characteristics and perioperative outcomes of patients undergoing ETSA (n= 50).

Variables	ESTA(n=50)
Gender(male/female)	27/23
Age, mean (SD), years	41.66 ± 9.70(27-62)
BMI, mean (SD)	24.82 ± 3.83
Diameter of the largest tumor, mean (SD), mm	8.68 ± 4.38(3-20)
Extent of surgery, n(%)	
Hemithyroidectomy with CND	23(46)
Bilateral thyroidectomy with CND	27(54)
Bleeding amount, mean (SD), mL	
Hemithyroidectomy(n=23)	13.48 ± 3.44
Bilateral thyroidectomy(n=27)	18.33 ± 5.27
Operative time,mean (SD), min	
Total	118.06 ± 33.81
Hemithyroidectomy with CND	93.61 ± 21.78
Bilateral thyroidectomy with CND	137.39 ± 27.76
Central lymph nodes (Metastatic/Retrieved), mean (SD)	
Total	2.16 ± 2.80/10.62 ± 5.08
Hemithyroidectomy with CND(n=23)	1.96 ± 3.10/8.96 ± 5.46
Bilateral thyroidectomy with CND(n=27)	2.33 ± 2.68/12.04 ± 4.03
Complications, n (%)	
Transient hypoparathyroidism	(4/27) 14.8%
Transient RLN palsy	(2/50)4.00%
Hematoma	(1/50)2.00%
Chyle leakage	(1/50)2.00%
Bleeding	(0/50)0%
Infection	(0/50)0%
Postoperative drainage volume, mean (SD), ml	
Total	90.16 ± 46.44
The day of surgery	11.06 ± 12.80
Postoperative day one	60.20 ± 35.10
Postoperative day two	18.90 ± 22.48
Postoperative hospitalization, mean (SD), days	2.84 ± 0.81
VAS pain score, mean (SD)	1.56 ± 0.61
Cosmetic satisfaction, mean (SD)	4.28 ± 0.67
THYCA-QOL(Average transformed score)	10.23 ± 5.33

CND, central neck dissection;, recurrent laryngeal nerve; SD, standard deviation; VAS, visual analog scale; THYCA-QOL, Thyroid Cancer-specific Quality of Life.

No statistical comparisons were conducted between ETSA and other surgical approaches, as the data from external studies were used for descriptive reference only.

## Result

**Figure 2:**
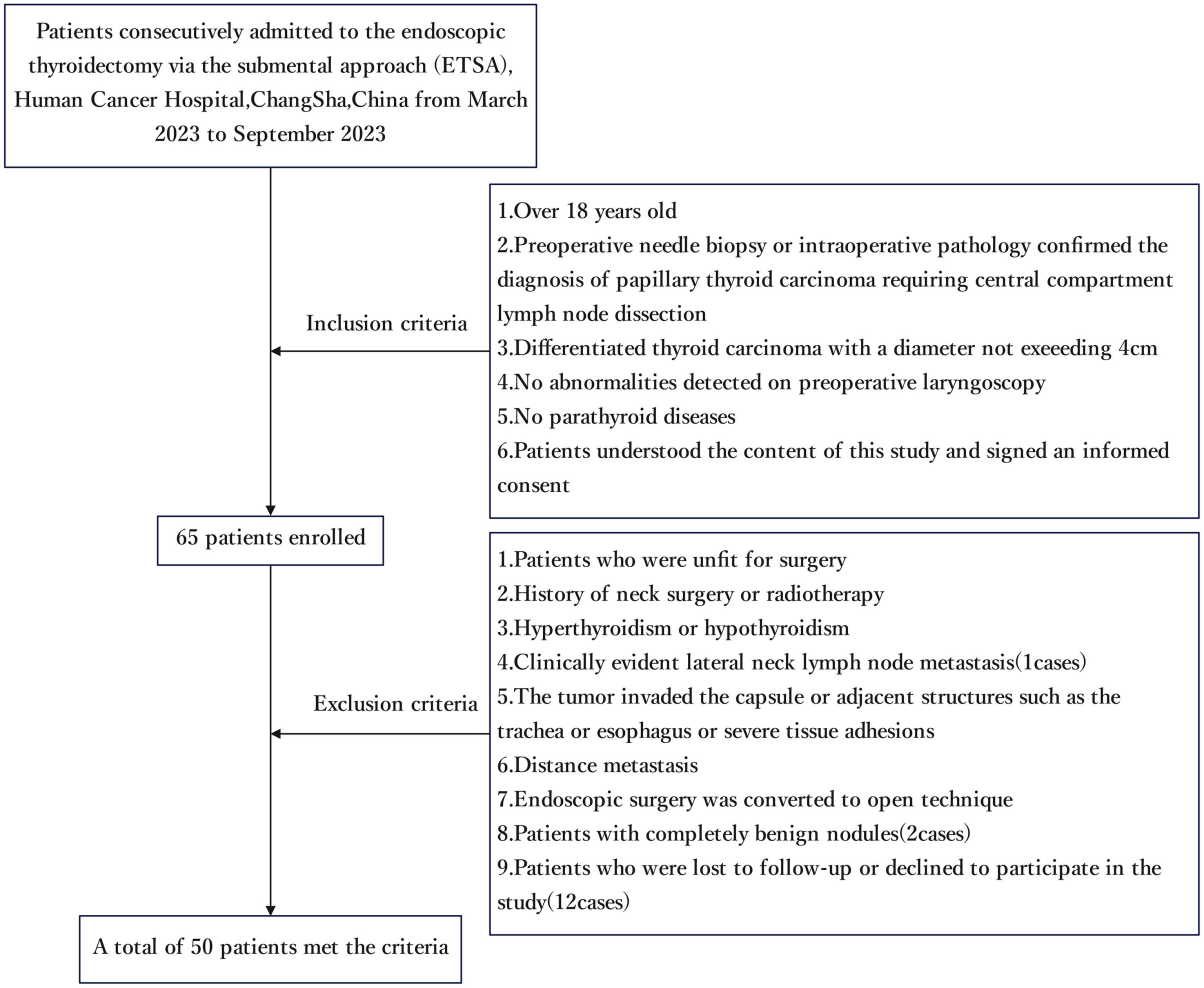
Flow diagram shows the process of study selection.

A total of 50 patients (27 males and 23 females; mean age 41.66 ± 9.70 years; mean BMI 24.82 ± 3.83 kg/m²) were included in this study. The average of the largest tumor diameters (one per patient) was 8.68 ± 4.38 mm. (**Figure 2** and **Table 1**).

Among them, 23 patients underwent unilateral lobectomy with central neck dissection (CND), and 27 patients underwent total thyroidectomy with CND. The mean operative time was 93.61 ± 21.78 minutes for unilateral procedures and 137.39 ± 27.76 minutes for total thyroidectomy, the overall mean operative time across all cases was 118.06 ± 33.81minutes. The mean number of metastatic/total lymph nodes dissected was 1.96 ± 3.10/8.96 ± 5.46 for unilateral cases and 2.33 ± 2.68/12.04 ± 4.03 for bilateral cases. The overall mean metastatic/total lymph node count was 2.16 ± 2.80/10.62 ± 5.08.

The mean drainage volume was 11.06 ± 12.80 mL on the day of surgery, 60.20 ± 35.10 mL on postoperative day one, and 18.90 ± 22.48 mL on postoperative day two, with a total mean drainage volume of 90.16 ± 46.44 mL. The average postoperative hospital stay was 2.84 ± 0.81 days. The mean VAS pain score at 48 hours postoperatively was 1.56 ± 0.61. The mean cosmetic satisfaction score was 4.28 ± 0.67 (scale 1–5, with higher scores indicating greater satisfaction), and the mean quality of life score (THYCA-QoL) was 10.23 ± 5.33 (scale 0–130, with lower scores indicating better QoL) (**Table 1**).

Postoperative complications included 4 cases (14.8%) of transient hypoparathyroidism (The rate of transient hypoparathyroidism was calculated among the 27 patients who underwent total thyroidectomy) and 2 cases (4.00%) of transient RLN palsy, all of which resolved within six months. In addition, 1 case (2.00%) of postoperative hematoma and 1 case (2.00%) of chyle leakage were observed. During a mean follow-up period of 22 months, no patients exhibited recurrence or distant metastasis (**Table 1**).

## Discussion

Endoscopic thyroid surgery has evolved into a diverse field, with various approaches offering unique advantages and facing distinct limitations (11–13, 15). Techniques such as trans-axillary, trans-breast/chest, and TOETVA have been widely adopted, each aiming to minimize visible scars, reduce surgical trauma and enhance oncological outcomes (7, 16–18). However, none of these methods achieves perfection across all dimensions.

In clinical practice, we observed that the ETSA represents a potential breakthrough. This approach appears to strike the balance among oncological thoroughness, minimal trauma, cosmetic outcomes and surgical feasibility. Its innovative design not only addresses the limitations of existing techniques but also sets a new benchmark for achieving comprehensive outcomes in thyroid surgery.

Oncological thoroughness is a cornerstone of thyroid cancer surgery, as inadequate resection or lymph node dissection may lead to recurrence and compromise long-term outcomes (19–23). Central neck lymph node metastasis is particularly common among Chinese patients with differentiated thyroid carcinoma (DTC), prompting Chinese guidelines to recommend prophylactic CND for all DTC cases, regardless of clinical nodal statu (23).

Both ETSA and TOETVA share a vertical trajectory, allowing direct top-to-bottom dissection of the central neck (16, 24–26). This orientation facilitates complete exposure of the central compartment and supports thorough lymph node clearance. When compared with our previously conducted TOETVA procedures performed by the same surgical team, ETSA achieved comparable CND outcomes. In our prior TOETVA cohort, the mean number of dissected central lymph nodes was 10.91 ± 6.47 (27)and 11.91 ± 7.61 (28), respectively, which is similar to the 10.62 ± 5.08 nodes observed with ETSA in this study (**Table 2**).

**Table 2:** Comparison of central lymph node yield among different surgical approaches for thyroidectomy.

Surgical methods	Hemithyroidectomy	Bilateral thyroidectomy	Total
ETSA	8.96±5.46	12.04±4.03	10.62±5.08
TOETVA (our previous published series)			11.91±7.61.Wen et al. (28)
			10.91±6.47.Zhou et al. (27)
Trans-axillary	3.3±2.7.Xu et al. (29)		
	6.36±4.75.Chen et al. (5)		
	5.13±2.26.Sun et al. (30)		
	3.94±2.86.Park et al. (31)		
	4.1±2.43.Cho et al. (32)		
Trans-chest/breast	6.34±4.10.Sun et al. (33)	6.4±2.1.Qu et al. (34)	
	3.14±1.75.Zhan et al. (26)	10.70±3.72.Wang et al. (35)	
	7.5±4.5.Xie et al. (36)		
	4.79±1.51.Yu et al. (37)		
COT	5.83±3.71.Sun et al. (33)	10.71±5.17.Sun et al. (38)	
	4.4±3.4.Xu et al. (29)	12.0±3.0.Yu et al. (39)	
	5.84±1.44.Jin et al. (40)		
	4.91±2.43.Sun et al. (30)		

In contrast, both trans-axillary and trans-breast/chest approaches access the central compartment obliquely from lateral or inferior planes and are anatomically constrained by the clavicle and sternum. These structural barriers may limit exposure and restrict the extent of lymph node clearance (29, 41, 42) (**Table 2**).

Notably, the trans-axillary approach is primarily used for unilateral thyroidectomy with ipsilateral central neck dissection, and published studies report mean central lymph node dissection numbers ranging from 3.3 ± 2.7 to 6.36 ± 4.75 for unilateral procedures (5, 29–32)—lower than those achieved with ETSA in similar unilateral cases. For the trans-breast/chest approach, reported central lymph node yields range from 3.14 ± 1.75–7.5 ± 4.5 for unilateral (26, 33, 36, 37) and 6.4 ± 2.1–10.70 ± 3.72 for bilateral procedures (34, 35), also below the results achieved with ETSA in this study.

Although COT offers direct anatomical access, published data report central lymph node yields of 4.4 ± 3.4–5.84 ± 1.44 for unilateral (29, 30, 33, 40) and 10.71 ± 5.17–12.0 ± 3.0 for bilateral dissections (38, 39)—figures that are comparable to or slightly lower than those achieved with ETSA. This may be partially attributed to the limited exposure angle in COT when dissecting the lower paratracheal and deep pretracheal regions, particularly in patients with short necks or deep surgical fields. In contrast, the submental approach offers a top-down perspective, providing more favorable access to these areas and potentially allowing for more comprehensive dissection (16, 24–26) (**Table 2**).

Taken together, these findings highlight that ETSA, like TOETVA, provides superior access to the central neck and enables comprehensive CND. Its ability to achieve lymph node yields exceeding those of other endoscopic thyroidectomy techniques—and even COT—underscores its effectiveness in the oncological management of thyroid cancer.

Minimizing surgical trauma is a key consideration in endoscopic thyroid surgery, as it directly impacts patient recovery, postoperative pain, and overall satisfaction. Among various endoscopic approaches, the ETSA demonstrates distinct anatomical advantages. As the approach closest to the surgical field, ETSA requires the shortest operative pathway and the least flap dissection, minimizing tissue disruption and surgical trauma.

In comparison, due to the necessity of performing the procedure in the oral vestibule, TOETVA may cause compression around the lips during surgery, leading to postoperative discomfort in the lip area (11, 40, 43). Similarly, during the establishment of the surgical tunnel, TOETVA requires partial detachment of the mentalis muscle, which may result in postoperative discomfort in the mentum region (12). Trans-axillary and trans-breast/chest approaches, while effective in avoiding visible neck scars, involve extensive flap dissection (24, 44), leading to increased surgical trauma.

The reduced trauma of ETSA is reflected in several postoperative indicators. In our study, the mean pain VAS score at 48 hours postoperatively was 1.56 ± 0.61, which was lower than that observed in our previous TOETVA cohort (2.08 ± 0.71) (27) (**Table 3**).

**Table 3:** Comparison of postoperative recovery indicators across different thyroidectomy approaches.

Surgical methods	VAS	Postoperative hospitalization (days)	Postoperative drainage volume (ml)
ETSA	1.56±0.61	2.84±0.81	90.16±46.44
TOETVA	2.5±0.1.Lee et al. (45)	4.46±0.80.Sun et al. (33)	175.75±54.21.Sun et al. (33)
	1.80±0.09.P et al. (13)	3.3±0.76.Luo et al. (11)	135.9±63.5.Luo et al. (11)
	2.08±0.71.Zhou et al. (27)	3.21±0.49.Jin et al. (40)	86.56±71.28.Zhou et al. (27)
	2.35±0.38.Liu et al. (46)	4.18±0.97.Liu et al. (46)	102.84±66.15.Zhou et al. (27)
	3.1±1.5.Shen et al. (47)	3.25±1.17.Zhou et al. (27)	168.35±77.25.Liu et al. (46)
Trans-axillary	3.2±0.7.Y et al. (48)	4.81±1.51.Lee et al. (49)	154.7±42.9.Xu et al. (29)
	2.31±1.61.Cho et al. (50)	4.2±1.2.Zhang et al. (42)	143.7±89.5.Lee et al. (49)
	2.60±0.94.Cho et al. (32)	3.75±0.81.Lee et al. (51)	168.7±44.4.Zhang et al. (42)
	2.07±0.79.Lee et al. (51)	4.56±1.11.Lee et al. (52)	144.35±51.64.Lee et al. (51)
	2.11±0.74.Lee et al. (52)		165.23±60.23.Lee et al. (52)
	2.6±1.1.Arora et al. (53)		
Trans- chest/breast	3.08±1.08.Zhu et al. (44)	4.27±0.95.Sun et al. (33)	169.48±55.67.Sun et al. (33)
	3.79±3.66.Liu et al. (46)	4.0±3.0. Liao et al. (54)	175.4±36.6.Zhang et al. (42)
	2.9±1.6.Shen et al. (47)	3.73±0.72.Zhan et al. (26)	144.60±57.07.Liu et al. (46)
	2.8±1.2.Arora et al. (53)	3.36±1.28.Liang et al. (55)	
		3.3±0.7.Qu et al. (34)	
COT	2.7±1.0.Yu et al. (39)	3.7±1.4.Yu et al. (39)	95.3±30.1.Xu et al. (29)
	2.7±1.9.Ünlü et al. (56)	4.18±1.02.Sun et al. (33)	117.44±46.18.Sun et al. (33)
	4.1±1.0.Qu et al. (57)	3.16±0.48.Jin et al. (40)	

When compared with other published data, the pain score for ETSA also falls below reported ranges for various endoscopic and open approaches: 1.80 ± 0.09 to 3.1 ± 1.5 for TOETVA (13, 45–47), 2.07 ± 0.79 to 3.2 ± 0.7 for trans-axillary (32, 48, 50–53), 2.8 ± 1.2 to 3.79 ± 3.66 for trans-breast/chest approaches (44, 46, 47, 53), and approximately 2.7 ± 1.0 to 4.1 ± 1.0 for COT (39, 56, 57).

While postoperative drainage volume is often used as a surrogate indicator for surgical trauma, an interesting observation emerged in our data. Compared with values reported in other studies, ETSA demonstrated significantly lower postoperative drainage volumes than those typically seen in transoral, trans-axillary, trans-breast/chest approaches and even COT. However, when compared with our previously conducted TOETVA procedures 86.56 ± 71.28 to 102.84 ± 66.15 (27), no significant difference in drainage volume was found (**Table 3**).

We hypothesize that this may be because drainage volume primarily reflects exudation from the thyroid bed, which is more influenced by the surgeon’s technique and intraoperative hemostasis rather than by the surgical approach itself. In this study, the primary surgeon had performed over 3,000 TOETVA procedures prior to initiating ETSA, with extensive experience in endoscopic thyroid surgery. This high level of surgical proficiency likely contributed to the consistently low drainage volumes observed in both the TOETVA and ETSA cohorts. These findings suggest that drainage volume may not accurately represent the intrinsic invasiveness of a given surgical approach, and further comparative studies are needed to clarify its validity as a trauma-related endpoint.

In contrast, postoperative hospital stay provides a more holistic reflection of surgical trauma, recovery speed, and patient confidence. Although thyroidectomy is often performed as a day surgery in some hospitals—particularly in Western countries—our ETSA group demonstrated a shorter hospital stay when compared with other studies involving standard inpatient management. The average postoperative stay for ETSA was 2.84 ± 0.81 days, which was lower than previously reported durations for various approaches: 3.21 ± 0.49–4.46 ± 0.80 days for TOETVA (11, 33, 40, 46), 3.75 ± 0.81–4.81 ± 1.51for trans-axillary (42, 49, 51, 52), 3.3 ± 0.7–4.27 ± 0.95 days for trans-breast/chest (26, 33, 34, 54, 55), and 3.16 ± 1.02–4.18 ± 0.48 days for COT (33, 39, 40).

Although we observed no significant difference in postoperative drainage volume between ETSA and our previous TOETVA cohort, the hospital stay was still shorter in the ETSA group (2.84 ± 0.81 vs. 3.25 ± 1.17 days) (27). This difference likely reflects faster postoperative recovery and reduced discomfort associated with the submental approach. Conversely, patients undergoing TOETVA may experience greater postoperative tension and soft tissue soreness (12, 13), particularly in the perioral and chin regions, which may lead to a more cautious attitude toward discharge.

These advantages align well with the principles of Enhanced Recovery After Surgery (ERAS) (58), which advocate for minimizing surgical stress, reducing hospital stay, and promoting early return to function. By facilitating faster recovery, reducing pain, and supporting earlier discharge without compromising safety, ETSA represents a trauma-conscious approach that integrates effectively with modern ERAS-based perioperative care pathways.

Endoscopic thyroidectomy is primarily sought after for its ability to avoid the visible neck scars associated with COT. In COT, the surgical incision is located on the anterior neck, making it difficult to conceal with clothing and often leading to psychological distress and social stigma. This concern transcends cultural and regional differences, significantly impacting patients’ QoL and mental well-being (3, 53, 59). Consequently, cosmetic outcomes are among the key factors influencing patients’ preference for endoscopic thyroidectomy.

The submental region is commonly used in plastic surgery procedures, such as liposuction and neck lifts, due to its ability to conceal surgical incisions. ETSA follows the same principle, utilizing a 0.5–1 cm incision hidden behind the chin. In a normal posture, this scar is barely noticeable, particularly in male patients, where facial hair provides additional coverage, further enhancing postoperative cosmetic satisfaction (**Figures 1C, D**). In our study, patients assessed their cosmetic satisfaction on a scale from 1 (“very dissatisfied”) to 5 (“very satisfied”). At six months postoperatively, the mean cosmetic satisfaction score was 4.28 ± 0.67, indicating a high level of patient satisfaction. Notably, no patients expressed significant concerns regarding visible scarring or noticeable surgical marks on the neck.

Beyond cosmetic outcomes, reduced surgical trauma associated with ETSA plays a crucial role in enhancing patients’ overall postoperative QoL. Given that DTC patients generally has an excellent prognosis, with most patients achieving long-term survival, treatment considerations now extend beyond oncological safety to include functional and psychological well-being (60, 61). Minimizing visible scars and surgical discomfort contributes significantly to better postoperative adaptation and patient satisfaction (3).

In this study, the mean THYCA-QoL (62) score was 10.23 ± 5.33 (lower scores indicating better QoL), reflecting favorable postoperative experiences among ETSA patients.

Upon analyzing domain-specific results, we found that ETSA achieved comparable or better scores across all measured domains compared to TOETVA (63), with no domains performing worse. Notably, the overall mean THYCA-QoL score for ETSA (10.23 ± 5.33) was lower than that observed with TOETVA (12.42 ± 9.37) (63), indicating a potential trend toward improved postoperative quality of life in the ETSA group.

When compared to COT, ETSA demonstrated consistent advantages in postoperative QoL as measured by the THYCA-QoL questionnaire. In our analysis of transformed domain scores, the overall mean score for ETSA was lower (10.23 ± 5.33) than that reported for COT (15.33 ± 10.04), indicating a potential trend toward better overall postoperative well-being. In another study (64) that applied raw domain scores, ETSA similarly showed favorable results across most dimensions. Notably, in both studies-regardless of scoring method-the ETSA group reported markedly lower scores in the ‘scar’ domain, reinforcing its superior cosmetic outcomes (**Table 4**). While scar-related concerns may not always rank among the most significant determinants of QoL, as noted by Sanabria et al. in a systematic review (65), the markedly lower scores observed in the scar domain still highlight ETSA’s superiority in meeting patients’ aesthetic expectations.

**Table 4:** Comparison of postoperative quality of life scores (THYCA-QoL) among ETSA, TOETVA, and COT.

Domain	ESTA		TOETVA		COT	
	Raw scores	Transformed scores	Raw scores	Transformed scores (63)	Raw scores (64)	Transformed scores (63)
Neuromuscular	1.21±0.32	7.11±10.60		8.37±13.05	1.48±0.60	12.50±13.35
Voice	1.33±0.51	11.00±16.87		12.16±17.33	1.57±0.68	15.81±21.69
Concentration	1.23±0.53	7.66±17.70		7.84±12.75	1.32±0.58	13.97±18.62
Sympathetic	1.21±0.40	7.00±13.37		13.73±18.58	1.28±0.57	13.60±18.83
Throat/mouth problems	1.30±0.37	10.00±12.22		14.64±16.37	1.40±0.60	15.60±15.79
Psychological	1.44±0.47	14.50±15.80		14.90±17.11	1.32±0.58	17.34±16.94
Sensory	1.37±0.45	12.33±14.84		12.55±17.61	1.17±0.38	19.00±20.80
Problems with scar	1.10±0.46	3.33±15.28		4.31±15.25	1.77±0.81	14.71±22.13
Felt chilly	1.24±0.43	8.00±14.24		10.59±19.39	1.53±0.72	16.42±21.47
Tingling hands/feet	1.12±0.33	4.00±10.83		5.10±14.09	1.29±0.59	8.09±16.48
Gained weight	1.48±0.64	16.00±21.33		17.25±23.91	1.05±0.24	21.32±28.31
Headache	1.24±0.51	8.00±17.08		8.63±16.39	1.35±0.60	11.03±18.62
Less interest in sex	1.72±0.57	24.00±18.90		31.37±27.39	1.33±0.58	19.85±25.13
Average		10.23±5.33		12.42±9.37		15.33±10.04

These findings collectively underscore the cosmetic and quality-of-life advantages associated with ETSA. By reducing visible scarring and minimizing postoperative discomfort, ETSA significantly enhances patient satisfaction and overall well-being. This dual benefit - favorable cosmetic outcomes combined with improved THYCA-QoL scores - highlights the potential of ETSA in better addressing patients’ aesthetic expectations and psychosocial needs following thyroid surgery. However, it should be noted that the comparisons between ETSA, TOETVA, and COT in this study involved different primary surgeons, which may introduce operator-related bias. To address this limitation and more rigorously evaluate postoperative quality of life across different techniques, future studies are planned to directly compare ETSA, TOETVA, and COT outcomes within the same surgical team.

Surgical feasibility is a critical factor in evaluating new surgical techniques. Although ETSA was initially developed to address anatomical limitations that hinder TOETVA, subsequent clinical practice revealed several additional advantages that enhance its practicality and surgical applicability.

One key distinction between ETSA and TOETVA lies in incision classification. While TOETVA involves a Class II incision (66), ETSA restores the incision classification to Class I, similar to COT. This theoretical advantage may contribute to a lower risk of surgical site infection. In our study, no prophylactic antibiotics were administered perioperatively, and importantly, no cases of postoperative surgical site infection were observed. These findings suggest that ETSA may offer a safer alternative in terms of infection control.

Furthermore, the submental incision avoids the rigid contour of the mandible, facilitating a more straightforward and efficient specimen extraction process. In contrast, TOETVA poses a risk of compressing the mental nerve during specimen removal (12), which may lead to postoperative discomfort or nerve-related complications (40, 67). ETSA eliminates this concern, providing a safer and more practical alternative for surgical specimen retrieval.

In addition to infection control and safer specimen handling, ETSA demonstrated low complication rates in critical postoperative outcomes. Among the 50 patients analyzed, transient hypoparathyroidism occurred in 14.8% of cases, and transient RLN palsy occurred in 4.00%. No cases of permanent hypoparathyroidism or RLN palsy were observed. Compared with complication rates reported for other approaches-including TOETVA (transient hypoparathyroidism 0-50%, RLN injury 0-12.5% (68), trans-axillary (0-33.3%,0-15%) (68), trans-breast/chest (0-17.6% (69), 2.5-31.1% (70)), and COT (17.1-44.6%, 0-19.5%) (68), the complication rates observed with ETSA fall within the lower end of the reported ranges, further supporting its surgical safety.

ETSA also shows advantages in operative maneuverability and efficiency. By eliminating mandibular interference, ETSA significantly reduces the “chopstick effect” (11, 12) and improves instrument handling. It also circumvents the impact of thyroid cartilage prominence (11), allowing for a more straightforward and flexible dissection compared to TOETVA (71).

A recent meta-analysis by Hindawi et al. evaluated outcomes of the transoral endoscopic thyroidectomy submental vestibular approach (TOETSMVA), a hybrid technique combining transoral and submental access. Their pooled analysis demonstrated that TOETSMVA was associated with significantly shorter operative time, reduced hospital stay, greater lymph node yield, and lower rates of mandibular numbness compared to TOETVA (72). In our study, the mean operative time for unilateral thyroidectomy with CND using ETSA was 93.61 ± 21.78 minutes, and for total thyroidectomy with bilateral CND, it was 137.39 ± 27.76 minutes. These operative times were numerically shorter than those recorded in our previous TOETVA cohort (100.52 ± 16.47 minutes for unilateral and 156.05 ± 25.60 minutes for bilateral surgeries) (27).

These cumulative advantages position ETSA as a versatile and promising option within the expanding spectrum of endoscopic thyroidectomy. Notably, a recent network meta-analysis comparing diverse endoscopic approaches confirmed that while each technique has distinct strengths, none is universally superior across all outcomes (73). Against this backdrop, ETSA offers a well-balanced alternative that merits broader consideration in clinical decision-making.

ETSA offers a practical and economically favorable profile. Its shorter operative time and reduced hospital stay (observed in our cohort) may lower direct costs related to operating room use and inpatient care. ETSA requires only standard laparoscopic instruments, without the need for robotic platforms or specialized devices, making it accessible for centers already equipped for basic endoscopic surgery. Additionally, its Class I incision profile and absence of surgical site infections in our series suggest reduced infection-related costs. These features support ETSA as a cost-conscious, scalable technique suitable for broader clinical adoption beyond tertiary hospitals.

## Limitations

Despite the promising outcomes observed in this study, several limitations should be acknowledged:

First, this study is a single-center retrospective analysis, which inherently carries a risk of selection bias. The lack of randomization limits our ability to control for potential confounding factors. Larger-scale prospective studies are needed to validate the observed advantages of ETSA in more diverse patient populations.

Second, although the complication rates in our cohort were low, the sample size may not be sufficient to detect rare adverse events. Expanding the case volume across multiple institutions will improve the statistical power and generalizability of findings.

Third, the average follow-up duration was only 22 months, which may be insufficient to fully assess long-term oncological outcomes such as recurrence and metastasis. Ongoing follow-up is essential to confirm the durability and cancer control efficacy of ETSA.

Furthermore, while ETSA appears technically more accessible than TOETVA, this study was performed by surgeons with extensive TOETVA experience. This prior proficiency likely smoothed the transition to ETSA and may not reflect the experience of surgeons without similar backgrounds. Additional training data and multi-center evaluations will be important to understand the learning curve for first-time ETSA adopters.

Finally, this study represents an early case series designed to assess the feasibility, safety, and short-term outcomes of ETSA. Given the exploratory nature and limited sample size, no statistical comparison across surgical techniques was performed. All between-approach comparisons were descriptive and based on literature reports, which may be influenced by inter-study heterogeneity in patient characteristics, case complexity, and surgeon experience. These findings should therefore be interpreted cautiously and regarded as hypothesis-generating. Future retrospective and prospective controlled studies within the same surgical team are planned to quantitatively evaluate the comparative performance of ETSA, TOETVA, and conventional open thyroidectomy.

## Conclusion

ETSA effectively achieves a balance among cosmetic outcomes, minimal surgical trauma, oncological completeness, and procedural feasibility, providing a novel and promising option for endoscopic thyroidectomy. Although the preliminary outcomes are encouraging, this study is limited by its retrospective design, small sample size, and short follow-up duration. Further prospective, multicenter studies with longer follow-up are needed to validate the long-term oncological safety, functional outcomes, and broader clinical applicability of ETSA.

Abbreviations: ETSA, endoscopic thyroidectomy via the submental approach; COT, conventional open thyroidectomy; TOETVA, transoral endoscopic thyroidectomy vestibular approach; iPTH, intact parathyroid hormone; DTC, differentiated thyroid carcinoma; ERAS, Enhanced Recovery After Surgery; QoL, quality of life; CND, central neck dissection; RLN, recurrent laryngeal nerve; VAS, visual analog scale; THYCA-QOL, Thyroid Cancer-specific Quality of Life. transoral endoscopic thyroidectomy submental vestibular approach, TOETSMVA.

## Data Availability

The datasets presented in this study can be found in online repositories. The names of the repository/repositories and accession number(s) can be found in the article/supplementary material.

## Supplementary material

Supplementary Video 1 Extra-corporeal demonstration of workspace creation in endoscopic ETSA.
