# Supplementary material for: Endoscopic thyroidectomy via the submental approach: a balanced approach to aesthetics, safety, and recovery- a case series study
Source: Front Endocrinol (Lausanne). 2026 Jan 12;16:1715390. doi: 10.3389/fendo.2025.1715390 (PMC12832485; doi:10.3389/fendo.2025.1715390)
Supplement: Supplementary file 2 [file Table1.docx]

Supplementary Table 1. Definition of Postoperative complications

| Complication | Definition | Assessment Method | Criteria and Time Window |
| --- | --- | --- | --- |
| Transient hypoparathyroidism | Postoperative hypocalcemia with low PTH resolving within 6 months | Serum calcium and PTH measurement | Postoperative hypocalcemia (calcium < 8mg/dl) with low PTH (<13 pg/mL), requiring supplementation but resolving within 6 months. |
| Permanent hypoparathyroidism | Postoperative hypocalcemia with low PTH persisting beyond 6 months | Serum calcium and PTH measurement | Persistent hypocalcemia (calcium <8mg/dl) with inappropriately low PTH (<13pg/mL), requiring supplementation beyond 6 months postoperatively. |
| Transient RLN palsy | Vocal cord mobility impairment recovering within 6 months | Laryngoscopy | Vocal cord paresis/paralysis confirmed within 1 week postop, recovering within 6 months |
| Permanent RLN palsy | Vocal cord mobility impairment persisting beyond 6 months | Laryngoscopy | Vocal cord paresis/paralysis confirmed at 6-month postoperative evaluation |
| Hematoma | Symptomatic blood collection requiring intervention | Clinical examination and ultrasound | Symptomatic neck swelling requiring intervention within 24-48h postoperative |
| Chyle leakage | Massive or milky drainage evident postoperatively | Drain output measurement and fluid analysis | The volume of drainage from the neck gradually increased after the operation. The color of the drainage fluid changed from a serum-like liquid to a milky and turbid liquid, or triglyceride > 1.129 mmol/L |
| Bleeding | Significant hemorrhage requiring reoperation | Clinical monitoring and surgical exploration | Active bleeding confirmed at reoperation within 24h postoperative |
| Infection | Wound infection within 30 days | Clinical examination and culture | Purulent drainage or diagnosed infection requiring treatment within 30 days |
